# Supplementary material for: Differential impact of environmental factors on airborne live bacteria and inorganic particles in an underground walkway
Source: PLoS One. 2024 Mar 21;19(3):e0300920. doi: 10.1371/journal.pone.0300920 (PMC10956794; doi:10.1371/journal.pone.0300920)
Supplement: S1 Table — The datasets include “temperature (T),” “humidity (H),” “atmospheric pressure (A),” “traffic pedestrians (TP),” “number of inorganic particles (Δ5: 1–5 μm),” and “number of live airborne bacteria”. (PDF) [file pone.0300920.s001.pdf]

Table S1. Dataset reused from our previous study (Dataset=60) including 'temperature (T)', 'humidity (H)', 'atmospheric pressure (A)', 'traffic pedestrians (TP)', 'number of inorganic particles (Δ5: 1-5μm)', 'number of live airborne bacteria'.

no.	Sampling Data	Time	temperature 'T' (°C)	humidity 'H' (%)	atmosspheric pressure 'A' (hPa)	traffic pedestrians 'TP' (person/10min)	number of inorganic particles (Δ5: 1-5μm)	number of live airborn bacteria estimated by SCD agar plate (CFU/m^3)*
1	20160502	0800 - 0810	15.5	37	1021	1330	61087	3.5
2	20160502	0840 - 0850	15.5	39	1022	794	58340	3.5
3	20160502	0920 - 0930	15.5	38	1021	523	57104	3.5
4	20160502	1000 - 1010	16	37	1021	445	51068	15.9
5	20160502	1040 - 1050	16.2	36	1021	661	53678	15.9
6	20160502	1120 - 1130	16.5	35	1020	717	74655	15.9
7	20160502	1200 - 1210	16.7	36	1019	807	94028	22.9
8	20160502	1240 - 1250	16.9	36	1019	830	76668	22.9
9	20160502	1320 - 1330	17.2	37	1020	820	77021	22.9
10	20160502	1400 - 1410	17.4	37	1019	859	71765	4.7
11	20160502	1440 - 1450	17.3	37	1019	890	151208	4.7
12	20160502	1520 - 1530	17.4	38	1019	954	83134	4.7
13	20160502	1600 - 1610	17.3	39	1018	897	88075	30.3
14	20160502	1640 - 1650	17.3	39	1018	976	83696	30.3
15	20160502	1720 - 1730	17	39	1018	1255	64343	30.3
16	20160502	1800 - 1810	17.1	40	1018	1422	68872	18.5
17	20160502	1840 - 1850	17.2	41	1020	1276	112266	18.5
18	20160502	1920 - 1930	17.4	42	1019	958	98839	18.5
19	20160601	0800 - 0810	19.3	57	991	1171	49935	15.7
20	20160601	0840 - 0850	19.6	58	991	1265	57493	15.7
21	20160601	0920 - 0930	20.2	59	991	617	45931	15.7
22	20160601	1000 - 1010	20.3	57	991	421	40577	11.3
23	20160601	1040 - 1050	20.7	54	991	602	36362	11.3
24	20160601	1120 - 1130	20.8	52	991	659	32278	11.3
25	20160601	1200 - 1210	20.5	53	991	914	36133	39.7
26	20160601	1240 - 1250	21.1	56	991	1261	65120	39.7
27	20160601	1320 - 1330	20.9	59	991	896	53113	39.7
28	20160601	1400 - 1410	20.5	59	991	1082	50571	1191.5
29	20160601	1440 - 1450	20.3	59	991	1022	54921	1191.5
30	20160601	1520 - 1530	18.8	55	991	917	37434	1191.5
31	20160601	1600 - 1610	19.5	57	991	854	59187	1617.4
32	20160601	1640 - 1650	19.7	57	991	1045	54886	1617.4
33	20160601	1720 - 1730	19.5	55	992	1378	51046	1617.4
34	20160601	1800 - 1810	18.9	51	991	1501	54914	23364.5
35	20160601	1840 - 1850	19.4	50	992	1082	59329	23364.5
36	20160601	1920 - 1930	19.2	48	992	925	56150	23364.5
37	20160705	0800 - 0810	22.1	51	1020	998	34805	546.4
38	20160705	0840 - 0850	22.8	53	1019	1106	52901	546.4
39	20160705	0920 - 0930	23.2	53	1019	563	37477	546.4
40	20160705	1000 - 1010	23.6	53	1019	430	26637	14.4
41	20160705	1040 - 1050	23.8	54	1018	655	31430	14.4
42	20160705	1120 - 1130	24.1	56	1018	647	32439	14.4
43	20160705	1200 - 1210	24.2	56	1018	701	37610	17.6
44	20160705	1240 - 1250	24.5	57	1018	732	43508	17.6
45	20160705	1320 - 1330	24.7	57	1018	714	46212	17.6
46	20160705	1400 - 1410	24.7	56	1018	600	61448	16.2
47	20160705	1440 - 1450	24.8	57	1018	706	44708	16.2
48	20160705	1520 - 1530	24.8	57	1018	674	34449	16.2
49	20160705	1600 - 1610	24.5	57	1018	809	40944	183.8
50	20160705	1640 - 1650	24.2	57	1018	865	50571	183.8
51	20160705	1720 - 1730	24	57	1018	1289	39530	183.8
52	20160705	1800 - 1810	23.5	58	1018	1444	38217	31.0
53	20160705	1840 - 1850	23.3	57	1018	1248	44111	31.0
54	20160705	1920 - 1930	23.1	58	1018	993	39906	31.0
55	20170715	0550 - 0600	24	65	1009	23	7946	11.4
56	20170715	0630 - 0640	25.5	68	1009	80	16594	11.4
57	20170715	0710 - 0720	25.7	68	1008	135	17304	11.4
58	20170715	2215 - 2225	25.8	68	1009	225	37787	32.6
59	20170715	2255 - 2305	26.4	71	1009	163	32136	32.6
60	20170715	2335 - 2345	26.5	72	1008	161	32490	32.6

*, Bacterial counts estimated by SCD (soybean-casein diseated) agar plate were calculated at intervals of 2 hours, and environmental factors measured during that time were linked to the same value.
