# Supplementary material for: Differential impact of environmental factors on airborne live bacteria and inorganic particles in an underground walkway
Source: PLoS One. 2024 Mar 21;19(3):e0300920. doi: 10.1371/journal.pone.0300920 (PMC10956794; doi:10.1371/journal.pone.0300920)
Supplement: S2 Table — Values show the number of OTUs. (PDF) [file pone.0300920.s002.pdf]

**Table S2.** Airborne bacterial flora dataset obtained from previous study

| Sampling Data | Read numbers (Phylum) | | | | | | | | | | | | | | | | | | | |
|---|---|---|---|---|---|---|---|---|---|---|---|---|---|---|---|---|---|---|---|---|
| | Acidobacteria | Alphaproteobacteria | Armatimonadetes | Bacteroidetes | Betaproteobacteria | Chlamydiae | Chlorobi | Chloroflexi | Cyanobacteria | Deinococcus-Thermus | Deltaproteobacteria | Dictyoglomi | Epsilonproteobacteria | FCB group | Firmicutes | Fusobacteria | Gammaproteobacteria | Planctomycetes | Spirochaetes | Tenericutes |
| 2016 May (8:00-10:00) | 295 | 124 | 0 | 30 | 79 | 0 | 0 | 0 | 30 | 0 | 53 | 0 | 55 | 0 | 236 | 0 | 10 | 64 | 0 | 0 |
| 2016 May (10:00-12:00) | 191 | 24 | 0 | 0 | 107 | 0 | 0 | 0 | 11 | 0 | 0 | 0 | 15 | 0 | 232 | 0 | 10 | 52 | 28 | 0 |
| 2016 May (12:00-14:00) | 23 | 112 | 0 | 0 | 0 | 0 | 0 | 0 | 54 | 0 | 0 | 0 | 11 | 0 | 267 | 0 | 26 | 0 | 26 | 0 |
| 2016 May (14:00-16:00) | 479 | 192 | 14 | 12 | 118 | 0 | 0 | 0 | 23 | 0 | 89 | 0 | 55 | 0 | 248 | 39 | 57 | 20 | 0 | 0 |
| 2016 May (16:00-18:00) | 325 | 123 | 0 | 0 | 10 | 0 | 0 | 0 | 164 | 0 | 0 | 0 | 12 | 0 | 938 | 0 | 221 | 0 | 0 | 0 |
| 2016 May (18:00-20:00) | 214 | 0 | 0 | 0 | 186 | 0 | 0 | 0 | 0 | 0 | 35 | 0 | 190 | 0 | 639 | 0 | 0 | 0 | 0 | 0 |
| 2016 June (8:00-10:00) | 229 | 146 | 0 | 0 | 31 | 0 | 0 | 0 | 0 | 0 | 27 | 0 | 0 | 0 | 270 | 0 | 0 | 10 | 0 | 0 |
| 2016 June (10:00-12:00) | 152 | 60 | 0 | 0 | 13 | 0 | 0 | 0 | 33 | 0 | 24 | 0 | 11 | 0 | 224 | 0 | 24 | 15 | 0 | 0 |
| 2016 June (12:00-14:00) | 215 | 21 | 0 | 0 | 247 | 0 | 0 | 0 | 0 | 0 | 108 | 0 | 67 | 0 | 109 | 0 | 72 | 48 | 0 | 0 |
| 2016 Julne (14:00-16:00) | 87 | 4279 | 0 | 0 | 11 | 0 | 0 | 0 | 88 | 0 | 26 | 0 | 48 | 0 | 279 | 0 | 0 | 0 | 70 | 0 |
| 2016 June (16:00-18:00) | 64 | 0 | 0 | 0 | 0 | 0 | 0 | 0 | 0 | 0 | 69 | 0 | 0 | 0 | 128 | 0 | 0 | 0 | 0 | 0 |
| 2016 June (18:00-20:00) | 433 | 168 | 0 | 42 | 186 | 0 | 0 | 0 | 0 | 0 | 42 | 0 | 0 | 19 | 106 | 0 | 0 | 0 | 0 | 0 |
| 2016 July (8:00-10:00) | 0 | 173 | 0 | 0 | 1427 | 45 | 0 | 0 | 225 | 0 | 1299 | 0 | 0 | 0 | 1359 | 0 | 0 | 0 | 0 | 0 |
| 2016 July (10:00-12:00) | 73 | 56 | 0 | 0 | 11 | 0 | 0 | 0 | 0 | 0 | 0 | 0 | 0 | 0 | 220 | 0 | 12 | 0 | 0 | 0 |
| 2016 July (12:00-14:00) | 3882 | 3458 | 0 | 186 | 724 | 0 | 35 | 367 | 113 | 0 | 381 | 199 | 79 | 39 | 6332 | 0 | 288 | 305 | 0 | 417 |
| 2016 July (14:00-16:00) | 220 | 366 | 0 | 0 | 0 | 0 | 0 | 0 | 12 | 0 | 174 | 0 | 0 | 0 | 76 | 0 | 241 | 0 | 0 | 0 |
| 2016 July (16:00-18:00) | 139 | 89 | 0 | 0 | 12 | 11 | 0 | 0 | 0 | 0 | 10 | 0 | 0 | 0 | 185 | 0 | 0 | 11 | 0 | 13 |
| 2016 July (18:00-20:00) | 2805 | 1507 | 74 | 110 | 1041 | 23 | 21 | 183 | 381 | 25 | 416 | 0 | 132 | 0 | 4174 | 89 | 3641 | 216 | 100 | 158 |
| 2017 July (5:50-6:50) | 8864 | 254 | 0 | 526 | 522 | 0 | 0 | 0 | 571 | 304 | 99 | 0 | 0 | 45 | 339 | 0 | 270 | 277 | 0 | 43 |
| 2017 July (6:50-7:50) | 9563 | 0 | 0 | 1033 | 75 | 0 | 0 | 11 | 0 | 99 | 0 | 0 | 0 | 74 | 457 | 0 | 179 | 0 | 0 | 0 |
| 2017 July (22:15-23:15) | 5680 | 367 | 0 | 817 | 0 | 0 | 0 | 0 | 2784 | 389 | 0 | 0 | 0 | 187 | 132 | 0 | 73 | 16 | 0 | 0 |
| 2017 July (23:15-24:15) | 6947 | 755 | 101 | 1419 | 441 | 0 | 0 | 0 | 1092 | 222 | 0 | 0 | 0 | 48 | 20 | 0 | 204 | 0 | 0 | 115 |
